# Effect of 1-methylcyclopropene on peel greasiness, yellowing, and related gene expression in postharvest ‘Yuluxiang’ pear

**DOI:** 10.3389/fpls.2022.1082041

**Published:** 2023-01-12

**Authors:** Dan Li, Xueling Li, Yudou Cheng, Junfeng Guan

**Affiliations:** ^1^ Institute of Biotechnology and Food Science, Hebei Academy of Agriculture and Forestry Sciences, Shijiazhuang, China; ^2^ School of Life Science and Engineering, Handan University, Handan, China

**Keywords:** pear, greasiness, chlorophyll, gene expression, wax, ethylene

## Abstract

‘Yuluxiang’ pear (*Pyrus sinkiangensis*) commonly develop a greasy coating and yellowing during storage. In this study, 1.0 μL L^–1^ 1-methylcyclopropene (1-MCP) was applied to ‘Yuluxiang’ pear to investigate its effects on fruit quality, peel wax composition, greasiness index, chlorophyll content, and the expression pattern of related genes during storage at ambient temperature (25°C). The results showed that 1-MCP treatment maintained higher fruit firmness and chlorophyll content, decreased respiration rate, and postponed the peak of ethylene production rate, lowered the greasy index of the peel. The main wax components of peel accumulated during storage, the principal ones being alkenes (C23, C25, and C29), fatty acids (C16, C18:1, and C28), aldehydes (C24:1, C26:1, and C28:1), and esters (C22:1 fatty alcohol-C16 fatty acid, C22:1 fatty alcohol-C18:1 fatty acid, C22 fatty alcohol-C16 fatty acid, C22 fatty alcohol-C18:1 fatty acid, C24:1 fatty alcohol-C18:1 fatty acid, and C24 fatty alcohol-C18:1 fatty acid), and were reduced by 1-MCP. 1-MCP also decreased the expression of genes associated with ethylene biosynthesis and signal transduction (*ACS1*, *ACO1*, *ERS1*, *ETR2*, and *ERF1*), chlorophyll breakdown (*NYC1*, *NOL*, *PAO*, *PPH*, and *SGR*), and wax accumulation (*LACS1*, *LACS6*, *KCS1*, *KCS2*, *KCS4*, *KCS10L*, *KCS11L*, *KCS20*, *FDH*, *CER10*, *KCR1*, *ABCG11L*, *ABCG12*, *ABCG21L*, *LTPG1*, *LTP4*, *CAC3*, *CAC3L*, and *DGAT1L*). There were close relationships among wax components (alkanes, alkenes, fatty acids, esters, and aldehydes), chlorophyll content, greasiness index, and level of expression of genes associated with wax synthesis and chlorophyll breakdown. These results suggest that 1-MCP treatment decreased the wax content of ‘Yuluxiang’ pear and delayed the development of peel greasiness and yellowing by inhibiting the expression of genes related to the ethylene synthesis, signal transduction, wax synthesis, and chlorophyll degradation.

## Introduction

Ethylene is an important hormone in the regulation of fruit ripening and senescence and is involved in the process of wax biosynthesis and chlorophyll breakdown. Endogenous ethylene increases total wax content (Li et al., 2022) and stimulates chlorophyll breakdown (Cheng and Guan, 2014) in postharvest fruits. In contrast, 1-methycycloprone (1-MCP), an ethylene action inhibitor, represses wax accumulation and chlorophyll breakdown (Curry, 2008; Cheng et al., 2012; Cheng and Guan, 2014; Li et al., 2019; Lv et al., 2020; Zhao et al., 2020; Li et al., 2022).

As one of the important components of the cuticle, wax plays an essential role in the postharvest life of fruit, including fruit surface characteristics, water loss, and fungal infection. Thus, surface wax can affect the quality of the fruit’s appearance. For example, alkane and fatty alcohols in wax can stabilize the three-dimensional structure of wax crystals, acting as inhibitors of peel greasiness in apples (Curry, 2008; Christeller and Roughan, 2016; Yang et al., 2017b), and also preventing the fruit surface from cracking in apples and cherries (Rios et al., 2015; Chai et al., 2020). Furthermore, aliphatic wax components (alkanes and aldehydes), when present at high concentrations, can form lamellar crystals and reduce fruit peel permeability in tomatoes and navel oranges (Leide et al., 2007; Leide et al., 2011; Liu et al., 2012; Petit et al., 2014; Wang et al., 2014).

The first step in fruit wax biosynthesis is the formation of a very long-chain fatty acid (VLCFA) (Samuels et al., 2008). In plastids, malonyl coenzyme A, as the donor of two-carbon units in VLCFA*_n_*_ ≤ 18_ elongation cycles, is synthesized through the catalysis of acetyl-coenzyme A carboxylase (CAC) (Liu et al., 2015; Liu et al., 2016). In addition, several enzymes and transporters are involved in the biosynthesis of VLCFA_20 ≤_ *_n_*_ ≤ 34_ and their derivatives, such as long-chain acyl-CoA synthetase (LACS), β-ketoacyl CoA synthetase (KCS), β-ketoacyl CoA reductase (KCR), fatty acyl-CoA reductase (CER), ATP-binding cassette transporter G protein (ABCG), lipid transporter protein (LTP), and LTPG1 (glycosylphosphatidylinositol-anchored lipid transfer protein) (Teerawanichpan and Qiu, 2010; Bernard and Joubès, 2013; Zhang et al., 2020a).

The greasiness of peel is a physiological characteristic related to wax metabolism in some fruits during storage. Excess grease may reduce the quality of their appearance, and therefore their commercial value. More detailed studies focused on apples have shown that peel greasiness is associated with fruit maturity and is more obvious in fruits with advanced ripeness, and that it is caused by the accumulation of fluid wax components resulting from a state change from solid to liquid on fruit surface wax (Christeller and Roughan, 2016; Yang et al., 2017a; Yang et al., 2017b; Zhang et al., 2019b). 1-MCP, aminoethoxyvinylglycine (AVG), and dynamic controlled atmosphere treatments have been shown to slow the process of grease accumulation on peel, to reduce mobile-phase wax components, and to down-regulate the expression of wax-related genes in apples during storage (Ju and Bramlage, 2001; Curry, 2008; Dong et al., 2012; Yang et al., 2017a; Klein et al., 2020a; Klein et al., 2020b).

Fruit yellowing is closely related to chlorophyll degradation and is controlled by a series of enzymes, such as chlorophyllase (CLH), pheophorbide a oxygenase (PAO), red chlorophyll catabolite reductase (RCCR), pheophytinase (PPH), non-yellow coloring 1 (NYC1), NYC1-like (NOL), and stay-green (SGR). Chlorophyll degradation in peel of pear is retarded by 1-MCP through depression of the expression of *PAO*, *NYC*, *NOL*, and *SGR* (Cheng et al., 2012; Cheng and Guan, 2014; Zhao et al., 2020).

However, the mechanism by which 1-MCP regulates wax biosynthesis and greasiness in pear fruits is little known, and the relationship between greasiness and yellowing in particular is unclear. In this study, ‘Yuluxiang’ pear (*Pyrus sinkiangensis*), a recently extended cultivar in China that has a tendency to develop greasiness and yellowing during storage, was chosen to investigate response to 1-MCP treatment, and to explore the molecular mechanism of the effect of 1-MCP on wax accumulation, greasiness, and yellowing during storage.

## Materials and methods

### Materials and treatments

‘Yuluxiang’ pear fruits were harvested from Shenzhou City, Hebei Province, during the commercial harvest period (i.e., on 30 August 2016) and transported to the laboratory within 2 h after harvest. The fruits chosen were of similar size (average fruit weight: 249.55 g) and showed no evidence of disease or mechanical or insect damage, and divided into two groups. One group was treated with 1-MCP (1.0 μL L^–1^) and the other with air (as the control) at 25 ± 1°C for 18 h (Dong et al., 2012); fruit were then stored at 25 ± 1°C.The fruit were sampled once a week to determine their fruit quality and wax content, and peel samples were immediately frozen with liquid nitrogen and then stored at –80°C for subsequent analysis.

### Methods

### Determination of fruit firmness and soluble solids content

Fruit firmness was measured with a fruit firmness tester (GY-4, Zhejiang, China) at the fruit equator, after peeling, and expressed in newtons (N). The soluble solids content (SSC) was measured with a digital refractometer (ATAGO PAL-1, Tokyo, Japan). Each experiment was carried out in triplicate, with five fruits used in each replicate.

### Determination of peel greasiness index

The greasiness index was determined according to DeLong et al. (2004). The greasiness of the peel was divided into four grades. No greasiness, mild greasiness, moderate greasiness, and severe greasiness were classed as grade 0, grade 1, grade 2, and grade 3, respectively. The greasiness index was calculated using the formula:


$$\begin{array}{l} \text{Greasiness index (GI) = }\Sigma \text{ (number of fruit in each grade }\times \text{ corresponding grade)}\\ \text{/(highest grade }\times \text{total number of fruit)} \end{array}$$

Each experiment was carried out in triplicate, with 10 fruits used in each replicate.

### Determination of chlorophyll content

Peel powder (0.5 g) was incubated in 15 mL of 80% (v/v) acetone solution at 25°C overnight. The chlorophyll (Chl) content was measured using the method described by Cheng et al. (2012), and the results were expressed as mg Chl g^–1^ fresh weight (FW). All measurements were performed in triplicate.

### Determination of rates of respiration and ethylene production

The fruit were sealed in desiccators for 30 min at 25 ± 1°C, then 10 mL of gas was extracted and the carbon dioxide content determined using an infrared analyzer (HWF-1, Jiangsu Jintan Instrument Manufacturing Co. Ltd, China) for the calculation of the rate of respiration. After fruit sealing for 3 h, 1 mL of gas was mixed and extracted for the determination of the rate of ethylene production by gas chromatography (GC) (9790 II, Zhejiang Fuli Analytical Instrument Co. Ltd, China). Each experiment was carried out in triplicate, with 10 fruits used in each replicate.

### Extraction and determination of wax components

The extraction of total wax was carried out using the method described by Wang et al. (2014). Fruit were divided among two containers and treated with chloroform for 30 s. Fruit extracted from the two containers were combined, and 400 μL of 0.5 μg μL^–1^ *N*-tetradecane (Accustadard Inc., New Haven, CT, USA) was added as an internal standard. The mixture was distilled using a rotary evaporator in a 40°C water bath and then dried with a slow nitrogen flow. The remaining substance was the total wax sample.

The wax sample was dissolved in pyridine, and incubated at 50°C for 30 min, following which 400 μL of *N*-*O*-trimethylsilyl trifluoroacetamide (TCI, Tokyo, Japan) was added and the sample incubated at 60°C for 40 min to enable a derivatization reaction to occur. The mixture was dried, and then re-dissolved in 2.5 mL of chloroform. The solution was filtered into a 1.5-mL headspace glass injection bottle with a filter membrane with a pore diameter of 0.22 μm, ready for wax determination.

The wax content was determined by gas chromatography–mass spectrometry (GC-MS). Wax components were separated in a capillary column (specification: 30 m × 0.25 mm, diameter × thickness). Helium was used as a carrier gas, and the flow rate setting was 1 mL min^–1^. The heating program of GC was as follows: the initial temperature was 70°C for 1 min; subsequently the temperature was increased to 200°C at a rate of 10°C per min, and then to 300°C at a rate of 4°C per min for 20 min. The mass spectrum program was as follows: the injection port temperature was 250°C, the transmission line temperature was 250°C, the ion source temperature was 230°C, and the electron impact (EI) ion source was 70 eV, with a mass spectrum scanning range of 50–650 *m*/*z*. The wax component was identified by the mass spectrum library NIST09, and was expressed as μg cm^− 2^.

### Ribonucleic acid extraction and real-time quantitative reverse transcription polymerase chain reaction analysis

RNA extraction was performed using the method reported by Gasic et al. (2004). The first strand of cDNA was synthesized by the Primescript™ RT reagent kit (Fischer Scientific, Waltham, MA, USA) with genomic DNA (gDNA) eraser. qRT-PCR was performed using an ABI 7500 quantitative PCR instrument (AppliedBiosystems, Foster City, CA, USA) and SYBR^®^Premix Ex Taq™ II kit (Tli RNaseH plus). The reaction system was as follows: 1 μL of forward primer and reverse primer (each at a concentration of 10 μmol L^–1^), SYBR 10 μL, Rox 0.4 μL, ddH_2_O 6.1 μL, and 12.5 ng of cDNA. The reaction procedure was 95°C for 15 s followed by 40 cycles of 95°C for 5 s and 60°C for 34 s. Relative gene expression was calculated according to the formula: 2^–ΔΔCT^ (Livak and Schmittgen, 2001). *ACT2* was used to standardize the transcript levels of the qPCR product in tested genes. Expression of each gene at day 0 was recorded as “1”. The specificity of primers was determined by the dissolution curve of qRT-PCR product. The primers of genes *LTPG1*, *LTP4*, *ACT2*, *ERS1*, *ETR2*, *CLH1*, *NYC1*, *RCCR*, *NOL*, *PPH*, and *SGR* were as reported in Wu et al. (2017); Li et al. (2017); Zhou et al. (2017), and Cheng and Guan (2014); the primers of the other genes were designed by Omega 2.0, and their sequences are shown in **Table S1**.

### Data analysis

All assays included three replicates, and the results were expressed as mean ± standard error. One-way ANOVA was used for the analysis of data difference. Duncan’s test (α = 0.05) was used for multiple comparisons in SPSS 20.0 software (IBM, Armonk, NY, USA). Principal component analysis (PCA) and heatmap analysis were conducted using Origin 2021 software (OriginLab, Northampton, MA, USA).

## Results

### 1-MCP treatment maintained higher fruit quality

**Figure 1:**
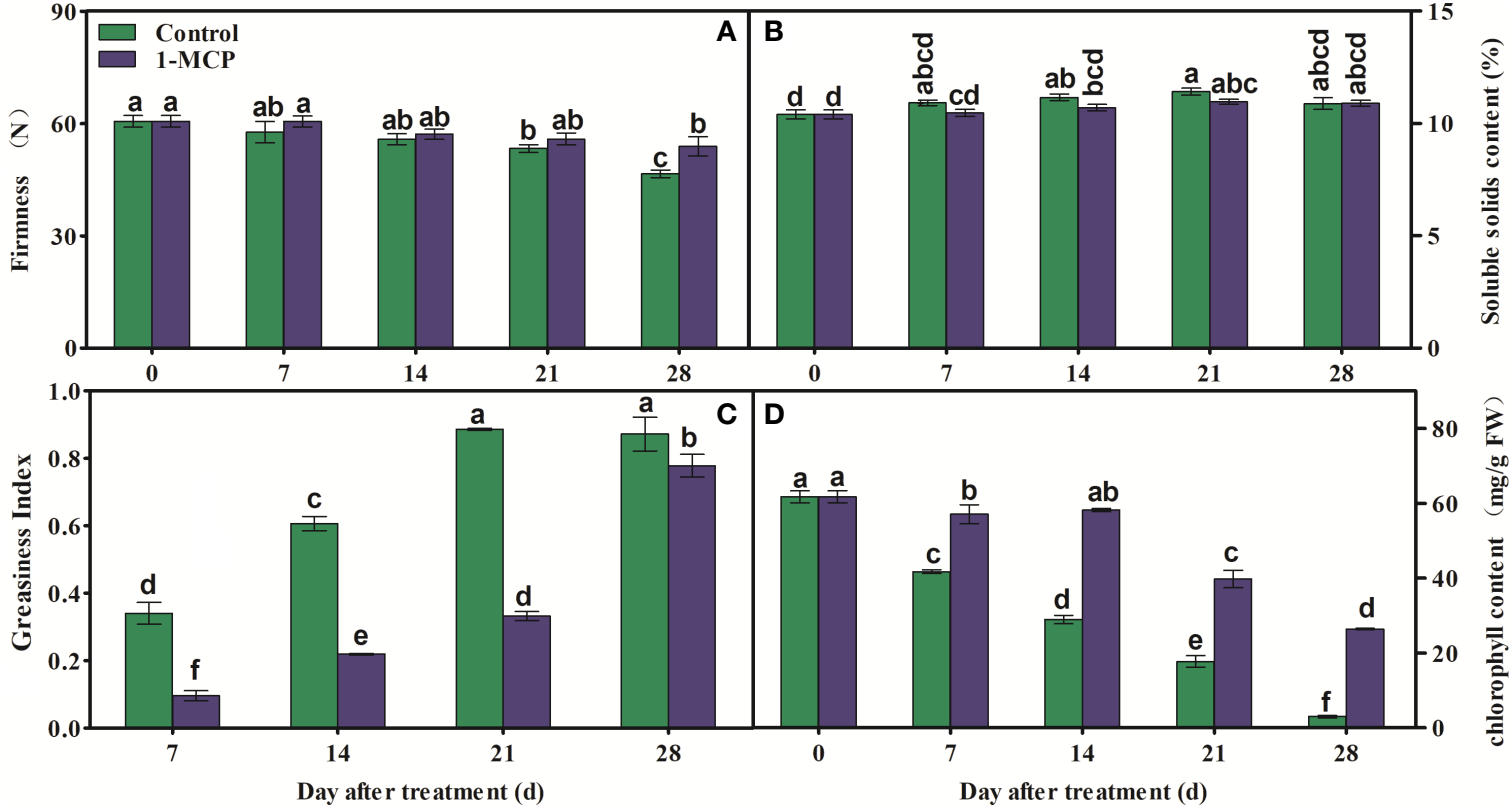
Effects of 1-MCP treatment on **(A)** fruit firmness, **(B)** soluble solids content (SSC), **(C)** greasiness index of peel, and **(D)** chlorophyll content in ‘Yuluxiang’ pear during storage. Data are mean ± SE (*n* = 3); values labeled with different letters represent a significant difference at *p*<0.05.

Fruit firmness clearly decreased in fruits subjected to both treatments after 21 days of storage, but it was higher in 1-MCP-treated fruit than in control fruit at day 28 (**Figure 1A**). There was no significant difference in SSC between treatments during storage (**Figure 1B**). In addition, GI was lower in 1-MCP-treated fruit than in the control fruit during storage (**Figure 1C**). The contents of chlorophyll decreased in both treatments, but the decrease was delayed by 1-MCP (**Figure 1D**).

### 1-MCP treatment decreased the rates of respiration and ethylene production

**Figure 2:**
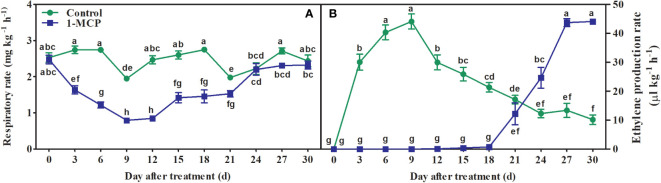
Effects of 1-MCP treatment on rates of **(A)** respiration and **(B)** ethylene production in ‘Yuluxiang’ pear during storage. Data are mean ± SE (*n* = 3); values labeled with different letters represent a significant difference at *p*<0.05.

The fruit showed an obvious ethylene production peak but no respiratory climacteric, although the lowest respiration rate was observed at day 6 in control fruit. The rate of respiration remained at a much lower level, and then slightly increased at day 24, in 1-MCP-treated fruit (**Figure 2A**). 1-MCP treatment significantly decreased the rate of ethylene production before day 18, and also delayed the appearance of its peak by 18 days (**Figure 2B**).

### 1-MCP treatment altered fruit wax composition and profile

The proportions of aliphatic wax components (alkanes, alkenes, fatty acid, ester, aldehydes, and fatty alcohols) and triterpenoids in ‘Yuluxiang’ pear were 39.4% and 51% at harvest (**Table S1**), respectively. Alkanes accounted for a higher proportion of wax than the other aliphatic components (**Table S1**). The above seven wax components accumulated in both treatments during storage, and the contents of alkenes, fatty acids, and esters were lower in 1-MCP-treated fruit than in control fruit (**Table 1**).

**Table 1:** Effects of 1-MCP treatment on the seven components of wax on the fruit surface of ‘Yuluxiang’ pear during storage.

Treatments	Day after treatment (d)	Wax component (µg cm^–2^)						
		Alkanes	Alkenes	Fatty acids	Esters	Aldehydes	Fatty alcohols	Triterpenoids
Control	0	26.93 ± 5.43 **d**	1.11 ± 0.02 **f**	1.67 ± 0.25 **d**	1.86 ± 0.24 **d**	5.92 ± 4.13 **c**	3.94 ± 0.62 **d**	53.68 ± 21.01 **cd**
	7	40.6 ± 8.05 **ab**	13.32 ± 3.92 **c**	10.47 ± 3.63 **b**	28.12 ± 5.92 **c**	25.97 ± 5.72 **a**	8.75 ± 1.5 **ab**	78.72 ± 25.4 **abc**
	14	36.08 ± 2.42 **bc**	16.19 ± 3.89 **c**	11.33 ± 0.58 **ab**	22.76 ± 15.46 **c**	24.05 ± 4.13 **a**	6.73 ± 0.36 **c**	73.87 ± 12.38 **abcd**
	21	31.67 ± 0.87 **cd**	20.78 ± 0.97 **b**	9.27 ± 0.67 **b**	42.03 ± 2.64 **b**	22.02 ± 0.04 **ab**	7.23 ± 0.3 **bc**	46.78 ± 1.69 **d**
	28	45.89 ± 3.81 **a**	35.8 ± 1.16 **a**	13.51 ± 0.43 **a**	53.55 ± 5.43 **a**	22.9 ± 1.56 **a**	6.44 ± 1.17 **c**	88.24 ± 0.39 **ab**
1-MCP	0	26.93 ± 5.43 **d**	1.11 ± 0.02 **f**	1.67 ± 0.25 **d**	1.86 ± 0.24 **d**	5.92 ± 4.13 **c**	3.94 ± 0.62 **d**	53.68 ± 21.01 **cd**
	7	35.37 ± 3.67 **bc**	4.87 ± 0.96 **e**	2.28 ± 0.24 **d**	9.27 ± 2.09 **d**	20.47 ± 0.88 **ab**	8.8 ± 1.13 **ab**	80.08 ± 4.64 **abc**
	14	29.17 ± 0.72 **cd**	3.74 ± 0.76 **ef**	2.1 ± 0.63 **d**	11.2 ± 3.34 **d**	16.72 ± 1.23 **b**	6.3 ± 0.85 **c**	67.63 ± 21.22 **abcd**
	21	39.46 ± 3.78 **ab**	8.95 ± 1.2 **d**	5.84 ± 1.09 **c**	21.91 ± 0.73 **c**	23.68 ± 2.02 **a**	9.62 ± 2.19 **a**	59.03 ± 18.94 **bcd**
	28	43.98 ± 1.58 **a**	15.94 ± 1.41 **c**	11.42 ± 0.48 **ab**	39.02 ± 5.36 **b**	24.11 ± 1.21 **a**	7.02 ± 0.41 **bc**	89.76 ± 3.02 **a**

1-MCP, 1-methylcyclopropene. Data are mean ± SE (*n* = 3); values in the same column labeled with different letters represent a significant difference at *p*<0.05.

**Figure 3:**
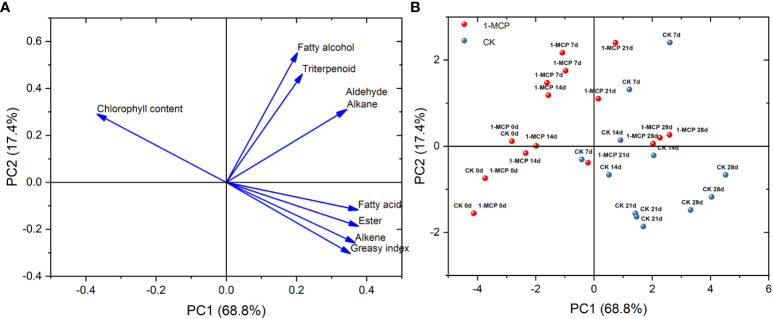
**(A)** Loading plot and **(B)** scoring plot of wax composition, greasiness index, and chlorophyll content based on PCA in control and 1-MCP-treated ‘Yuluxiang’ pear during storage.

PCA showed that the contribution of total variance to PC1 and PC2 reached 86.2%. Fatty acids, esters, alkenes, and greasiness index were located on the positive axis of PC1 and on the negative axis of PC2. Fatty alcohols, triterpenoids, aldehydes, and alkanes were located on the positive axes of PC1 and PC2. However, chlorophyll content was on the negative axis of PC1 and the positive axis of PC2 (**Figure 3A**).

The positions of the control at day 7 and day 14 were on the positive axis of PC1, while 1-MCP treatment at day 7 and day 14 loaded on the negative axis of PC1. The positions of the control at day 21 and day 28 were located on the negative axis of PC2, while the positions of 1-MCP treatment on day 21 and day 28 were mainly on the positive axis of PC2 (**Figure 3B**).

**Figure 4:**
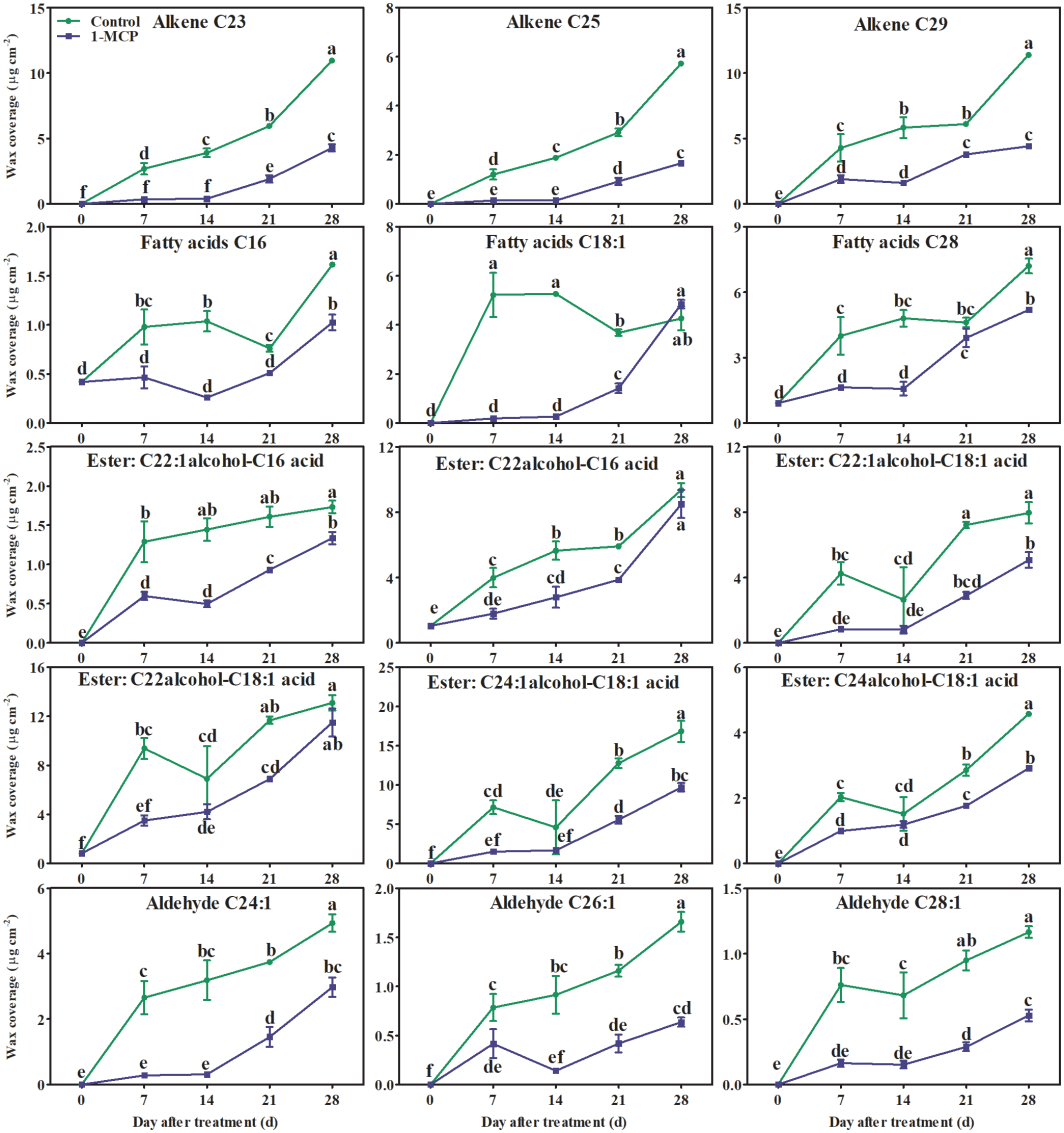
Effects of 1-MCP treatment on the principal components (alkenes, fatty acids, esters, and olefinic aldehydes) of wax on the surface of ‘Yuluxiang’ pear during storage. Data are mean ± SE (*n* = 3); values labeled with different letters represent a significant difference at *p*<0.05.

Alkanes and alkenes with carbon chains of C21–C31, three fatty acids (C16, C18:1, and C28), aldehydes (C24–C34), fatty alcohols (C21–C30), six esters (C22:1 fatty alcohol-C16 fatty acid, C22:1 fatty alcohol-C18:1 fatty acid, C22 fatty alcohol-C16 fatty acid, C22 fatty alcohol-C18:1 fatty acid, C24:1 fatty alcohol-C18:1 fatty acid, and C24 fatty alcohol-C18:1 fatty acid), and seven triterpenoids were all detected in the surface wax of ‘Yuluxiang’ pear fruits (**Figure S1**). Among them, one alkane (C29), three alkenes (C23, C25, and C29), three fatty acids and six esters (see above), five aldehydes (C24:1, C26:1, C28:1, C32, and C34), two fatty alcohols (C22 and C24), and 28-oxours-12-en-3-yl acetate were the main components of wax (Fig S1). The contents of the major components of wax, three alkenes (C23, C25, and C29), three fatty acids (C16, C18:1, and C28), six esters, and three aldehydes (C24:1, C26:1, and C28:1), showed an upward trend in both treatments during storage; however, they were reduced by1-MCP treatment to different degrees (**Figure S1** and **Figure 4**). No marked differences in the contents of alkane C29, aldehydes C32 and C34, fatty alcohols C22 and C24, and 28-oxours-12-en-3-yl acetate were found in either treatment during storage (**Figures S1**, **5**).

**Figure 5:**
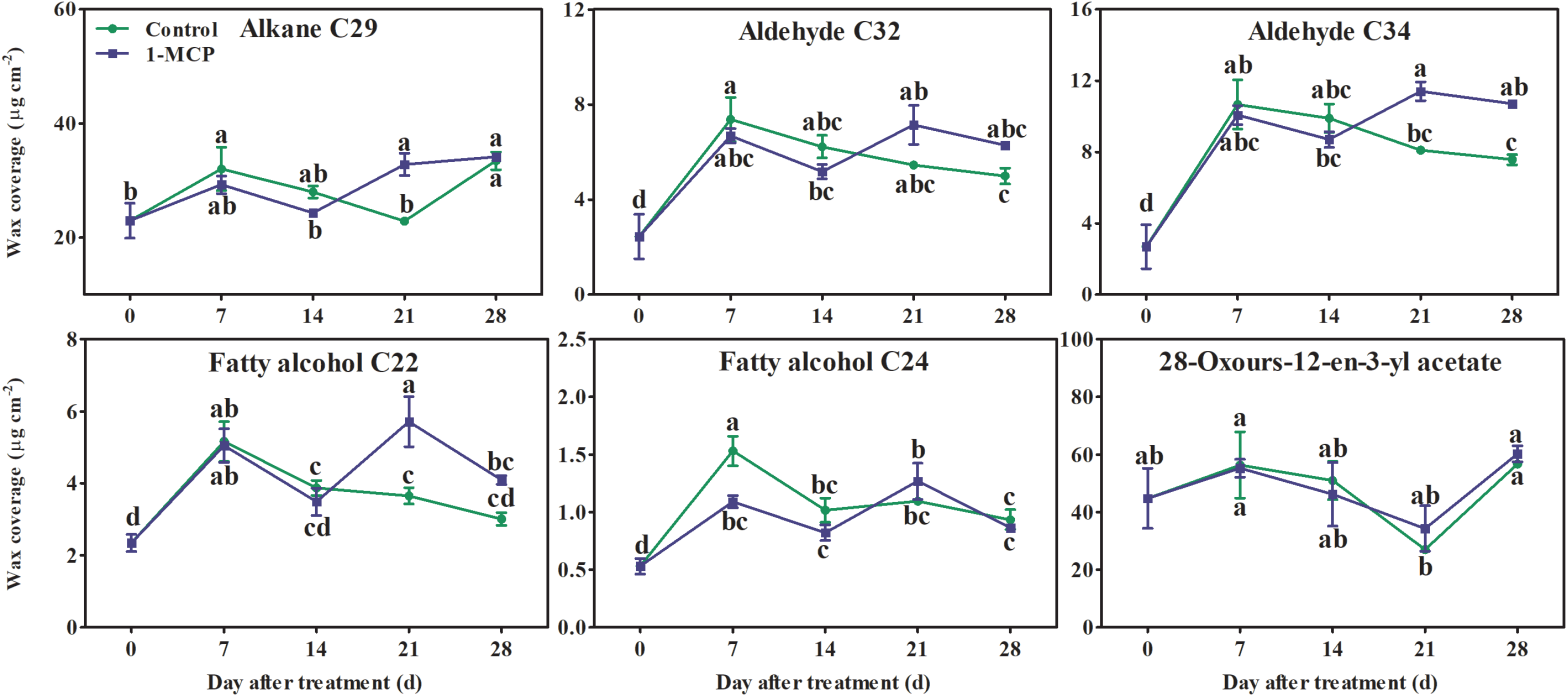
Effects of 1-MCP treatment on the principal components (alkanes, aldehydes, fatty alcohols, and triterpenoids) of wax on the surface of ‘Yuluxiang’ pear during storage. Data are mean ± SE (*n* = 3); values labeled with different letters represent a significant difference at *p*<0.05.

### 1-MCP treatment regulated the expression pattern of genes associated with ethylene synthesis and signaling

**Figure 6:**
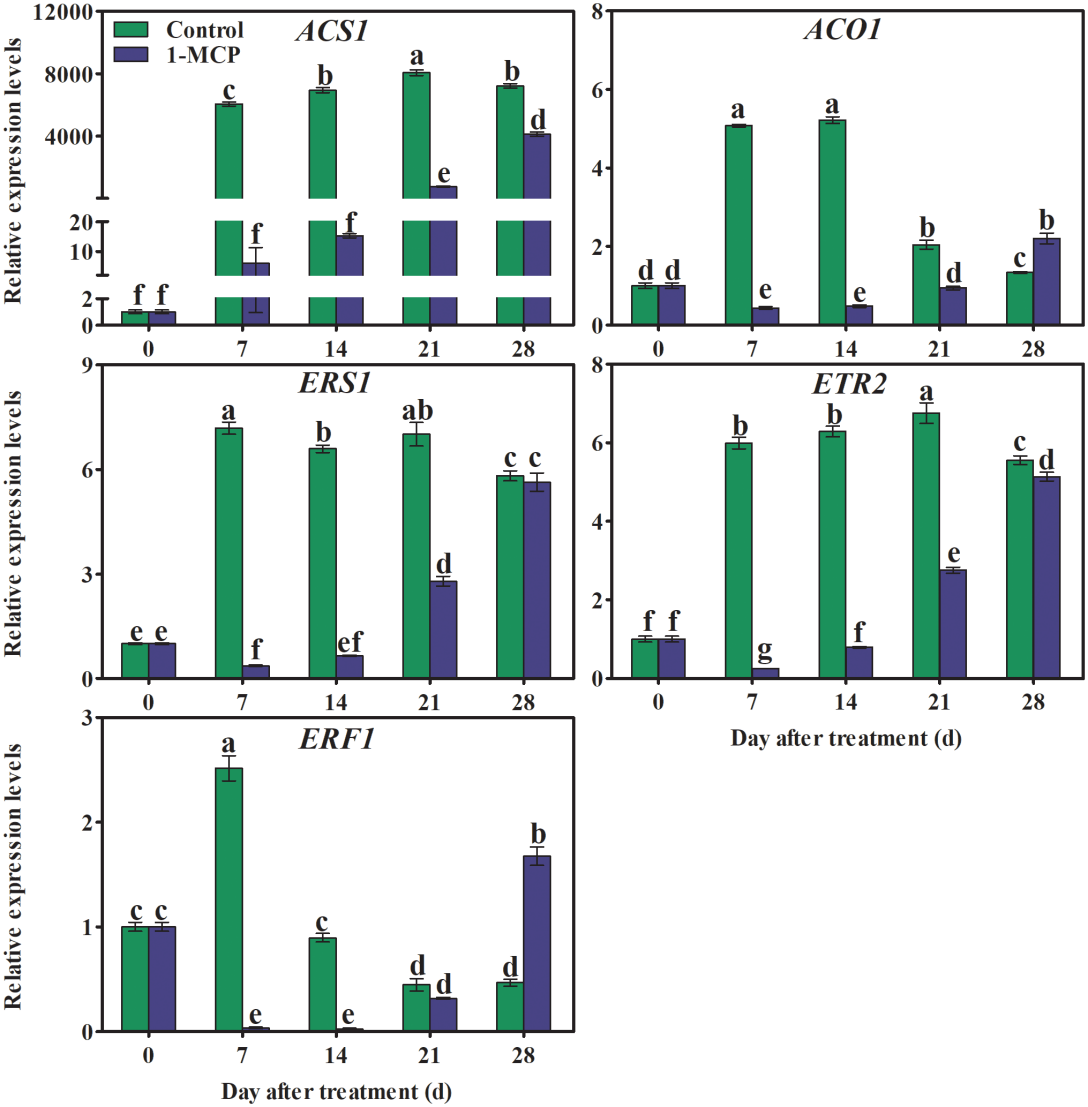
Effects of 1-MCP treatment on the expression levels of genes associated with ethylene biosynthesis (*ACS1* and *ACO1*), receptors (*ERS1* and *ETR2*), and signaling (*ERF1*) in the peel of ‘Yuluxiang’ pear during storage. Data are mean ± SE (*n* = 3); values labeled with different letters represent a significant difference at *p*<0.05.

The transcriptional levels of genes associated with ethylene synthesis (*ACS1*, *ACO1*) sharply increased during the first storage stage. Thereafter *ACS1* remained almost unchanged, while *ACO1* decreased in the control fruit, and both genes were down-regulated by 1-MCP. The expression in 1-MCP-treated fruit of genes encoding ethylene receptors (*ERS1*and *ETR2*) increased rapidly at first and then more slowly, before finally reducing. In control fruit, expression of *ERF1* quickly reached a maximum and then decreased before slowly increasing again. In comparison, peak expression of *ERF1* was delayed by 21 days in 1-MCP-treated fruit (**Figure 6**).

### 1-MCP treatment regulated the expression pattern of genes associated with chlorophyll degradation

**Figure 7:**
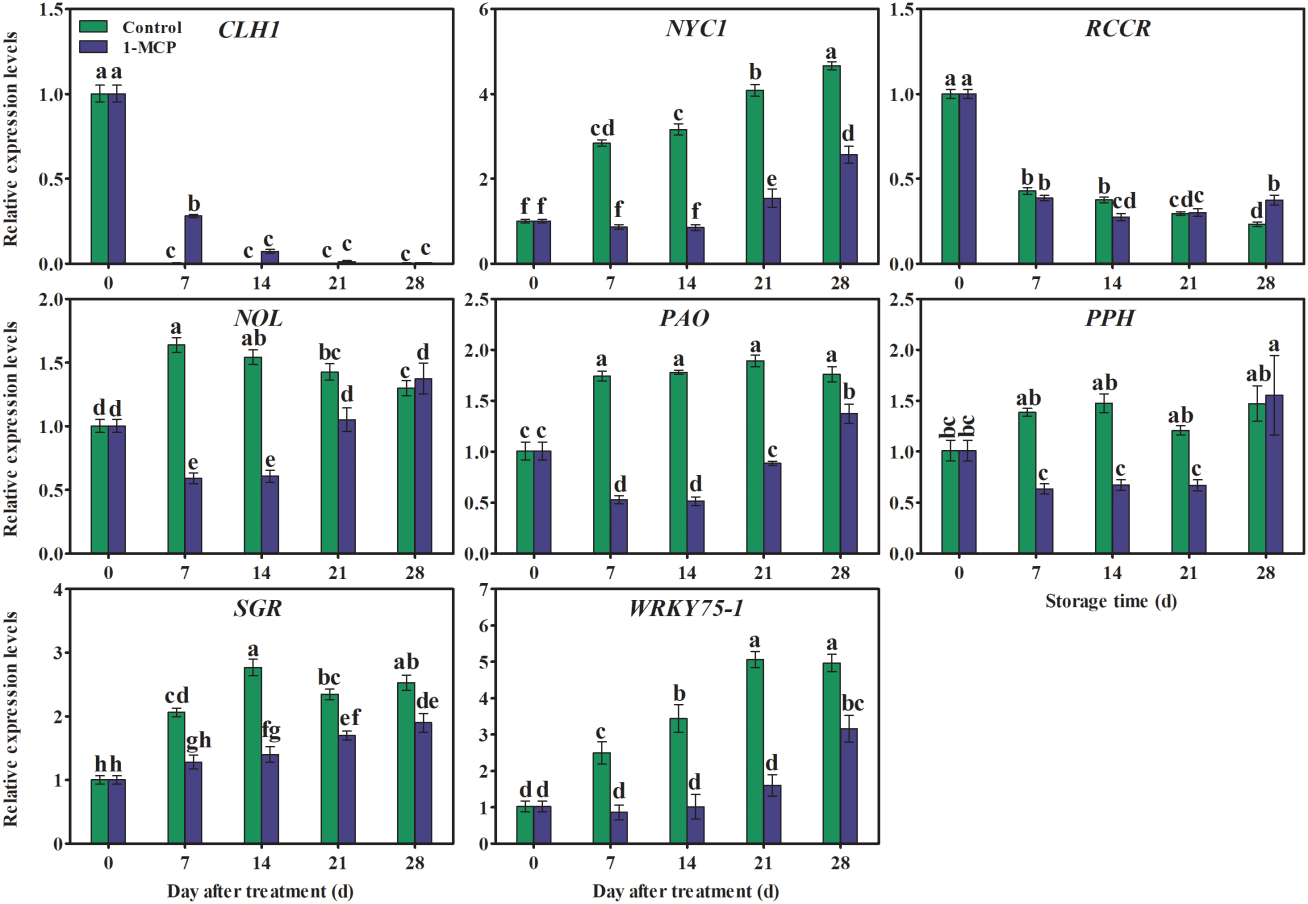
Effects of 1-MCP treatment on the expression pattern of genes associated with chlorophyll degradation in the peel of ‘Yuluxiang’ pear during storage. Data are mean ± SE (*n* = 3); values labeled with different letters represent a significant difference at *p*<0.05.

The expression of *NYC1*, *NOL*, *PAO*, *PPH*, and *SGR* increased rapidly during storage (with the exception of *NYC1*, which showed a gradual increasing trend as storage time increased) and was down-regulated by 1-MCP. In contrast, the relative expression levels of *CLH1* and *RCCR* decreased during storage. In 1-MCP-treated fruit, the expression of *CLH1* was higher at day 7, while the expression of *RCCR* was lower at day 14 and higher at day 28 (**Figure 7**).

### 1-MCP treatment regulated the expression pattern of genes associated with wax synthesis and transduction

The *CAC* gene is involved in the biosynthesis of VLCFA*_n_*_ ≤ 18_ in fruit wax. The present work showed that the expression of *CAC3* increased rapidly at first and then decreased. The expression levels of *CAC3L* gradually increased in control fruits, but were reduced in fruit treated with 1-MCP.

**Figure 8:**
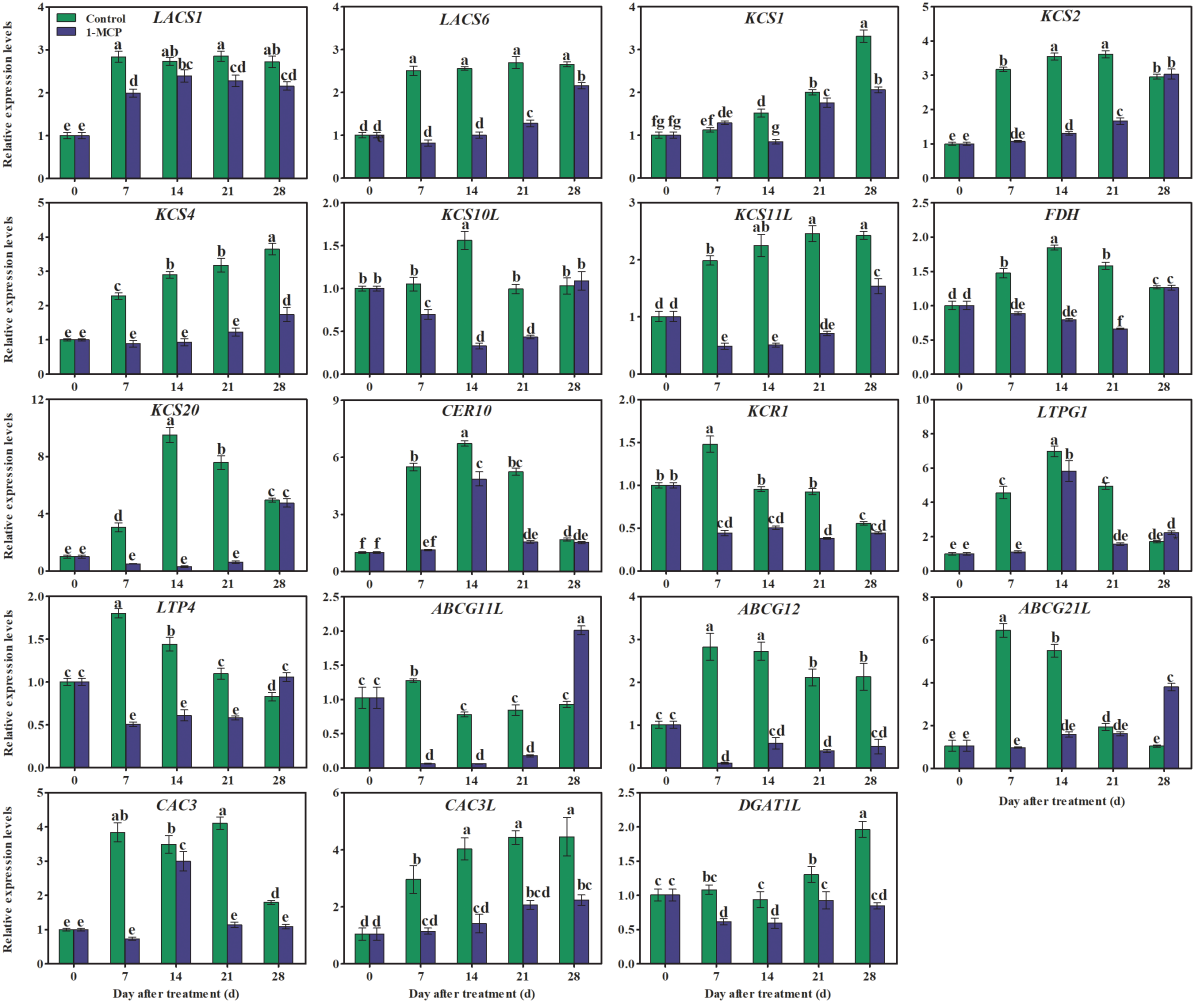
Effects of 1-MCP treatment on the expression pattern of genes associated with wax synthesis and transporter proteins in the peel of ‘Yuluxiang’ pear during storage. Data are mean ± SE (*n* = 3); values labeled with different letters represent a significant difference at *p*<0.05.

The genes *LACS*, *KCS*, *KCR*, *CER*, *ABCG*, *LTP*, and *LTPG* encode important enzymes or transporters associated with the biosynthesis of VLCFA_20 ≤_ *_n_*_ ≤34_. Here, the expression of *KCS1*, *KCS4*, and *KCS11L* showed an upward trend in control fruit, but the expression of these genes was down-regulated by 1-MCP treatment (**Figure 8**). In contrast, the expression of *LACS1*, *LTP4*, *KCR1*, *ABCG12*, *ABCG11L*, and *ABCG21L* rapidly increased at first and then declined, at first somewhat sharply and then more gradually in the control fruit. The expression levels of *KCS2*, *KCS10L*, *FDH(KCS)*, *KCS20*, and *LTPG1* peaked midway through the experiment (day 14) and were also down-regulated by 1-MCP (**Figure 8**).

### Correlation analysis between greasiness index, chlorophyll content, wax composition content, and expression levels of genes involved in wax synthesis and chlorophyll degradation

**Figure 9:**
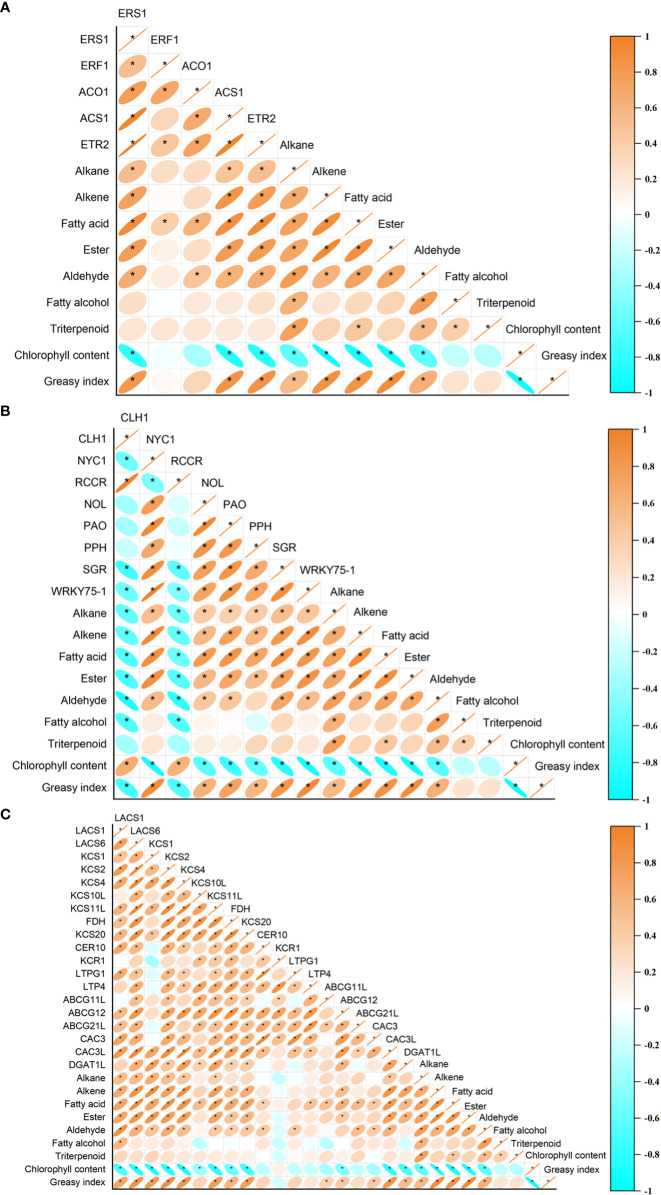
The relationships between peel greasiness index, chlorophyll content, and wax composition and **(A)** ethylene-related genes, **(B)** chlorophyll degradation genes, and **(C)** wax synthesis and transporter genes. The correlation significant level was set at P<0.05.

The expression levels of *ERS1*, *ACS1*, and *ETR2* showed a positive correlation with greasiness index and the contents in wax of alkanes, alkenes, fatty acids, esters, and aldehydes, but were negatively correlated with chlorophyll content (**Figure 9A**). In addition, the expression profiles of *ERF1* and *ACO1* were positively correlated with the fatty acid content of wax (**Figure 9A**). The expression levels of *CLH1* and *RCCR* showed a negative correlation with the greasiness index and the contents of fatty alcohols, aldehydes, esters, fatty acids, alkenes, and alkanes, but were positively correlated with chlorophyll content (**Figure 9B**). Expression levels of the *NYC1*, *NOL*, *PAO*, *PPH*, *SGR*, *LACS1*, *LACS6*, *KCS1*, *KCS2*, *KCS4*, *KCS11L*, *FDH*, and *KCS20* genes were positively correlated with the greasiness index, but the contents in wax of chemicals (such as aldehydes, esters, fatty acids, alkenes, and alkane) were negatively correlated with chlorophyll content (**Figures 9B, C**). In addition, there was no significant correlation between the expression of *PPH* and aldehyde content, or between the expression of *FDH* and *KCS20* and wax alkane content (**Figures 9B, C**). There was a positive correlation between the expression of three genes (*ABCG12*, *CAC3L*, and *DGAT1L*) and both greasiness index and the contents of some wax components (such as esters, fatty acids, and alkenes), but expression of the same three genes was negatively correlated with chlorophyll content (**Figure 9C**). Expression of the *CER10* and *LTPG1* genes was positively correlated with aldehyde content (**Figure 9C**). *LTP4* expression was positively correlated with fatty acid content (**Figure 9C**). Expression of *ABCG11L* was positively correlated with greasiness index and fatty acid content (**Figure 9C**). The relative expression levels of *ABCG21L* and *CAC3* showed a positive correlation with the contents of aldehydes and fatty acids, and expression of *CAC3* also showed a positive correlation with greasiness index (**Figure 9C**).

## Discussion

In the present work, 1-MCP treatment decreased the greasiness index and inhibited the decline of fruit firmness and content of chlorophyll (**Figures 1A, C**). The content of chlorophyll was negatively correlated with the greasiness index and with the composition of the wax mobile phase (i.e., the contents of alkenes, fatty acids, esters, and aldehydes) (**Figure 9A**). The degradation of chlorophyll was found to be accompanied by the accumulation of wax and increased peel greasiness in ‘Yuluxiang’ pear.

Ethylene increases the fatty acid and ester content of wax, and also promotes the formation of wax, in apple (Contreras et al., 2016; Yang et al., 2017b; Li et al., 2022), whereas 1-MCP has the opposite effects (Li et al., 2022). In this work, 1-MCP treatment inhibited the production of ethylene in fruit and delayed peak ethylene concentration (**Figure 2B**). A similar pattern was found in the expression of five ethylene-related genes (*ACS1*, *ACO1*, *ERS1*, *ETR2*, and *ERF1*) and 19 wax synthesis and transportation genes (*LACS1*, *LACS6*, *KCS1*, *KCS2*, *KCS4*, *KCS10L*, *KCS11L*, *FDH*, *KCS20*, *CER10*, *KCR1*, *LTPG1*, *LTP4*, *ABCG11L*, *ABCG12*, *ABCG21L*, *CAC3L*, *DGAT1L*, and *CAC3L*), and expression levels of these genes were also down-regulated by 1-MCP (**Figures 6**, **8**). In addition, the changes in the contents of total fatty acids, esters, and alkenes were also consistent with the pattern of expression of ethylene-related genes and wax synthesis genes (**Table 1**; **Figures 6**, **8**). Thus, it can be suggested that ethylene might be involved in regulation of the synthesis of wax in ‘Yuluxiang’ pear during storage.

It has been reported that the components of solid-phase wax on fruit surfaces, including alkanes and fatty alcohols, promote the morphogenesis of wax crystals (Wu et al., 2017; Yang et al., 2017b), while the components of mobile-phase wax, such as oleic acid and linoleic acid and their esters, are closely associated with peel greasiness in apples (Christeller and Roughan, 2016; Yang et al., 2017a; Yang et al., 2017b; Zhang et al., 2019b). In the present study, it was also found that the mobile-phase components (i.e., the three main fatty acids, six esters, three alkenes, and three aldehydes) accumulated during the development of peel greasiness in ‘Yuluxiang’ pear (**Figures 1**, **4**, **9**), and were reduced by 1-MCP (**Figure 4**). It has been reported that alkanes and fatty alcohols can contribute to the formation of wax crystals (Yang et al., 2017b). In this study, the proportions of alkanes and fatty alcohols were reduced in both control and 1-MCP-treated fruit, but their declines were retarded by 1-MCP (**Table S2**).

It has been shown that the *CAC1* and *CAC3* genes in apple, the *CAC* gene in the ‘Newhall’ orange mutant glossy, and two homologs of the *PbrCAC1* gene in ‘Yuluxiang’ pear are involved in the biosynthesis of VLCFA*_n_*_ ≤ 18_ in wax (Liu et al., 2015; Yang et al., 2017a; Wu et al., 2019). Here the expression levels of *CAC3* and *CAC3L* were consistent with the changes in the amounts of two fatty acids (C16 and C18:1) in fruit wax (**Figures 4**, **8**), and both genes were down-regulated by 1-MCP.

It has been reported that genes coding for fatty acid elongation enzymes (*LACS*, *KCS*, *KCR*, and *CER10*) and transport proteins (*ABCG* and *LTP*) are involved in the biosynthesis of C20–C34 fatty acids and their derivatives (Teerawanichpan and Qiu, 2010; Bernard and Joubès, 2013). The relatively high expression of *MdLACS2* and *MdKCS1* in apple, *SlCER6* in tomato, and *CsKCS1*, *CsABC*, and *CsLTP* in cucumber contributes to the accumulation of wax (Zhang et al., 2019a; Zhang et al., 2019c; Xiong et al., 2020; Zhang et al., 2020b). The *LACS1* gene, eight *KCS* genes (*KCS2*, *KCS4, KCS6*, *KCS9*, *KCS10*, *FDH1*, *KCS20*, and *CER60*), four *ABCG* genes (*CER5_1*, *CER5_2*, *WBC11_1*, and *WBC11_2*), and two *LTP* genes (*LTPG1* and *LTP4*) have all been linked to the accumulation of total wax and fatty acids in developing and ethephon-treated ‘Yuluxiang’ pear (Wu et al., 2019; Wu et al., 2020). In ‘Newhall’ navel orange, the levels of expression of two *KCS* genes (*KCS4* and *KCS2b*), two *ABCG* genes (*ABCG40* and *ABCG6*), and the *LTP1* gene were found to be correlated with the change in total wax content (Wan et al., 2020). In this study, lower quantities of three alkenes (C23, C25, and C29), C28 fatty acid, six esters, and three aldehydes were found in the surface wax of 1-MCP-treated fruit, and were also linked to the down-regulation of wax-related genes (**Figure 8**).

It is known that the protein DGAT1 possesses the structure domains of ester and diacylglycerol synthetase (Stöveken et al., 2005; Yen et al., 2005). The level of transcription of *DGAT1* mRNA was consistent with the content of the corresponding wax ester on the surface of pear fruits in cold storage (Wu et al., 2017). In this study, both the level of expression of *DGAT1L* and the ester content of peel were down-regulated by 1-MCP (**Figure 8**).

The process of ripening and senescence is delayed by 1-MCP treatment through inhibition of the ethylene signal and down-regulation of the expression of genes related to chlorophyll degradation (*RCCR2*, *NYC1*, *NYC3*, *NOL2*, *PPH*, *PAO*, *RCCR*, and *CLH*) in apple, pepper, and sweet cherry fruits (Lv et al., 2020; Du et al., 2021; Zhao et al., 2021). A study of the relationship between ethylene and fruit yellowing showed that 1-MCP delays chlorophyll degradation in ‘Comice’ pear by reducing ethylene production and the expression of chlorophyll catabolic signal genes, such as *PcPPH*, *PcNOL*, *PcSGR1*, and *PcPAO* (Zhao et al., 2020). In addition, 1-MCP inhibits the degreening induced by chlorophyll degradation in broccoli by down-regulating the expression of some chlorophyll degradation genes (Gómez-Lobato et al., 2012). In the present study, expression levels of chlorophyll degradation genes (*NYC1*, *NOL*, *PAO*, *PPH*, and *SGR*) were found to be lower in 1-MCP-treated fruit; as a result, chlorophyll content in 1-MCP-treated fruit remained high, and subsequent yellowing of the peel was delayed.

Correlation analysis suggested that chlorophyll content and associated gene expression were correlated with the expression of ethylene signal genes (*ACS1*, *ACO1*, *ERS1*, *ETR2*, and *ERF1*), and with the contents of major wax components and expression of related genes, which are closely related to the greasiness index (**Figure 9**).Thus, 1-MCP reduced the greasiness of ‘Yuluxiang’ pear during storage, by reducing the components of wax mobile-phase, and inhibited yellowing and the expression of chlorophyll breakdown pathway genes in peel, which are closely related to the ethylene signal function in ‘Yuluxiang’ pear.

## Conclusion

1-MCP (1.0 μL L^–1^) treatment significantly improved fruit firmness and chlorophyll content, reduced the components of mobile-phase wax (fatty acids, esters, alkenes, and aldehydes) and greasiness index, and also down-regulated the expression of genes associated with ethylene synthesis and signal (*ACS1*, *ACO1*, *ERS1*, *ETR2* and *ERF1*), wax synthesis and transduction (*LACS1*, *LACS6*, *KCS1*, *KCS2*, *KCS4*, *KCS10L*, *KCS11L*, *FDH*, *KCS20*, *CER10*, *KCR1*, *LTPG1*, *LTP4*, *ABCG11L*, *ABCG12*, *ABCG21L*, *CAC3*, *CAC3L*, and *DGAT1L*), and chlorophyll degradation (*NYC1*, *NOL*, *PAO*, *PPH*, and *SGR*). In this way, it repressed peel greasiness and yellowing in ‘Yuluxiang’ pear.

## Data availability statement

The raw data supporting the conclusions of this article will be made available by the authors, without undue reservation.

## Author contributions

JG: conceived and designed the experiments. DL and XL: performed the experiments. DL, XL and YC: analyzed the data. DL, JG, and YC wrote, reviewed and revised papers. All authors contributed to the article and approved the submitted version.
