# Supplementary material for: Effect of 1-methylcyclopropene on peel greasiness, yellowing, and related gene expression in postharvest ‘Yuluxiang’ pear
Source: Front Plant Sci. 2023 Jan 12;13:1082041. doi: 10.3389/fpls.2022.1082041 (PMC9878607; doi:10.3389/fpls.2022.1082041)
Supplement: Supplementary file 1 [file DataSheet_1.docx]

Supplementary Material

# Supplementary Figures and Tables

## Supplementary Figures


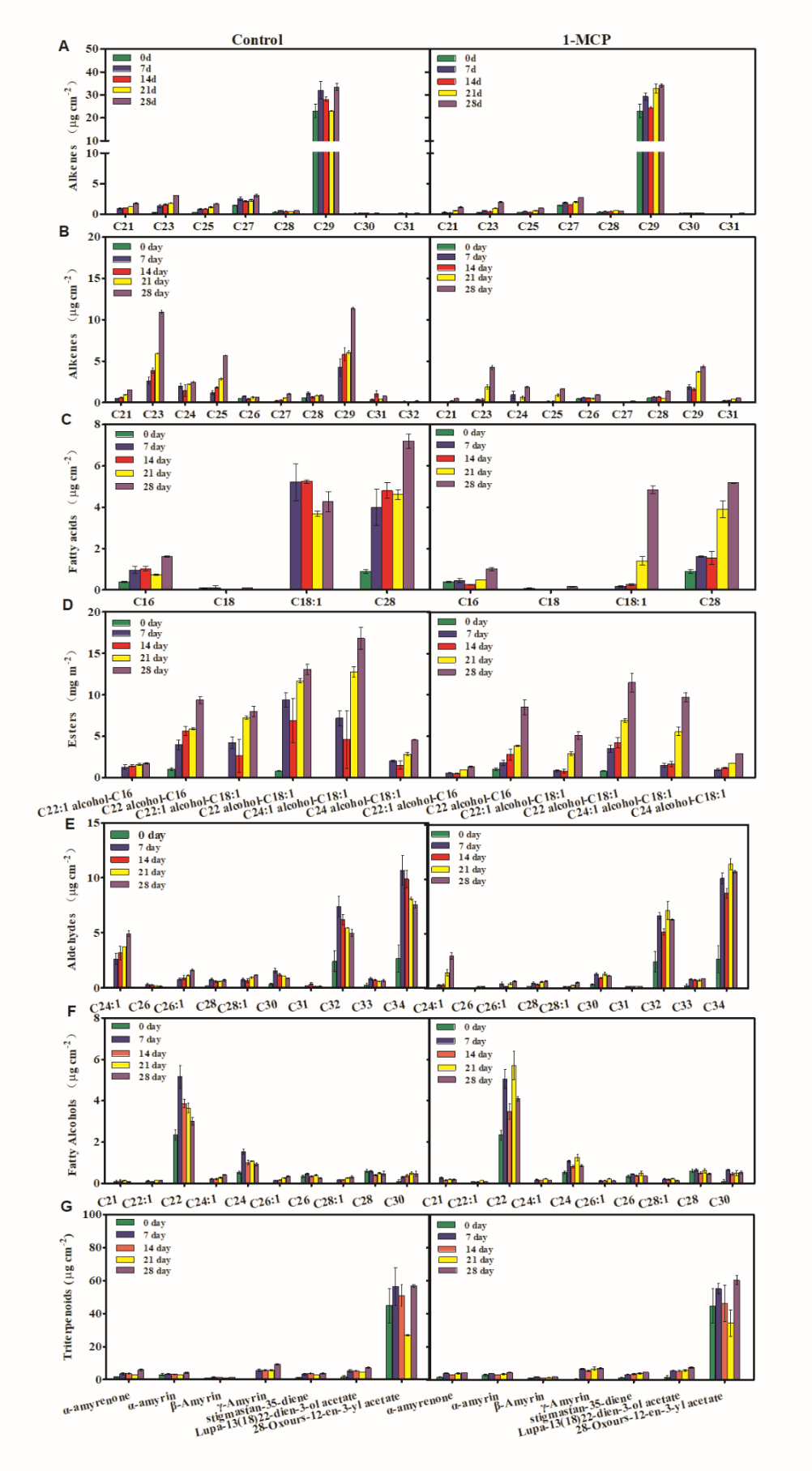


**Supplementary Figure 1.** Effects of 1-MCP treatment on profile of wax composition at fruit surface of ʻYuluixangʼ pear during storage. Data were mean ± SE (n=3). (A) Alkanes; (B) Alkenes; (C) Fatty acids; (D) Esters; (E) Aldehydes; (F) Fatty alcohols; (G) Triterpenoids.

## Supplementary tables

**Supplementary Table 1.** Primers of ethylene, wax related and chlorophyll degradation genes for quantitative real-time PCR analysis

| Gene | Accession No | Forward primer (5′-3′) | Reverse primer |
| --- | --- | --- | --- |
| *ACS1* | XM_018643584.1 | GAGATTGTACCCATTCACTGCA | AGGAGGTAGAGTTCGTTTCTGGT |
| *ACO1* | XM_009380726.2 | TCAGTGGCATCAATGGTAGTG | CATATTTTCCAGCACACTCAGTG |
| *ERS1* | AF386515 | CGTTGCATTCATCGTCAAACTG | TCGATGAACTTGCGCCAAATCG |
| *ETR2* | HM561909 | GATCCTGGAGAATCATCAGAGC | GCAGTTACAATGCAACCAAGC |
| *ERF1* | XM_009380720.2 | GCTTACCAACATGCATTGCGA | TGGGGTGTTTTTGTTTTCTGCT |
| *LACS1* | XM_009364839.1 | AGAAGGCGAGGATGGCAA | CCAAGCATTCGATTTCCAGG |
| *LACS6* | XM_009365944.2 | ATCGTTGATCATGCCTGCTC | CAGGCGTACAGTTGGAATATCC |
| *KCS1* | XM_009353114.2 | ATGGATGCTGCTTCTGTTCA | CTTGAGCTTGACGGACTGTAAG |
| *KCS2* | XM_009350716.1 | TCAAGGATGACGCTCTACAGGT | AACGCAATCTGCCACATCC |
| *KCS4* | XM_009337932.2 | CCTTGTCCAAAGATTTGATGG | ATAAGGCTTGACATTCGACTTGA |
| *KCS10L* | XM_009364715.2 | TCTGTTTACTTCATGTCACGCC | CGTCTCCTAAGCCTGATGAATT |
| *KCS11L* | XM_009349923.2 | CCTTGTTTTCCTATCCACCG | CTGGAACTGAAGATTGTCCTCTG |
| *KCS20* | XM_018646010.1 | TCTCGGTCACTTTGTGCTCC | GAAAAGCCAAGTTCTCGTCGTA |
| *FDH* | XM_009360773.2 | TTCTCAGTGAGGGTGCAAAGG | AGCACTGAACACCAGCACAAG |
| *KCR1* | XM_009360989.2 | TCAACTGATGGTTATGCCCG | GCCATGCGTCAACAACAGA |
| *CER10* | XM_018644500.1 | AAGCCCAGATGGAAATGGAG | GATGGAAGCGGCAACTATGA |
| *LTPG1* | XM_009374000.2 | CGGTGAAGGGGATAAGGGAG | AATCGGAGACAGTGGCGTTT |
| *LTP4* | XM_009373738.2 | TTTGGTGGTGGCCTTGAGC | TCCTGGCGATGCCGTTA |
| *ABCG11L* | XM_009339595.2 | AGCCGTATATTACTCTGCTCAGCT | GCTCAATACAAATGCTTACTCTCCT |
| *ABCG12* | XM_009376649.2 | GATCTAAGTGCGGTGCTTCC | GCCATGATCCTACCAGGCT |
| *ABCG21L* | XM_009357750.1 | AGGTTGTGTTGCTTCACATGA | CTTGCCGGAGATTTTATTCG |
| *CAC3* | XM_009371940.2 | CCATTGGCTGTGCTAATACATTAC | GCTTTTCAGCAGCCTTTGG |
| *CAC3L* | XM_009360561.2 | TGAGAGCCCTAGGGAAGGTAA | CACTCCACCTTTGACATTGG |
| *DGAT1L* | XM_009363362.2 | TCGACTCGAACGTGAACCAG | TCTTGAACAGCATTGACGGACT |
| *CLH1* | XM_009372504.3 | CCAAAGCCCTTGTTCATTGTTAC | CTGAGGTGCTACAGCTATGAATCC |
| *NYC1* | JN167997 | AATTTCTTCTTTCTGGAGATCGTGTAG | TGCATGTGTCAGGTTCTTGGA |
| *NOL* | JN167996 | CCGTGAGGCAATAAAAATGATG | CGCTTTGTTGCCCCGTAT |
| *PPH* | JN167999 | CGATTATGCTTGCTCCTCAAGG | AGGCCCCAAATAGGTTTCACC |
| *PAO* | JN167998 | AGCTAATGCCACCAAGCCTCC | AGCCATAGAACAGATCACGCTGG- |
| *SGR* | JN168000 | ATTCAACAAGGTGGAGTGCTGG | GCCGCTGTTGTTTTCCTGG |
| *RCCR* | JN168002 | TTCATAGACTTCCCCTACGTGTCG | GGAGAGTACAAGGCAAGAGCTGC |
| *WRKY75-1* | LOC103961435 | TCCAAAGTAGTAAATCATCGAATGG | TCACAGTTGGCGCACTAGTTC |
| *WRKY75-2* | LOC103935109 | GGAACCCAGCTCATCACGT | ATCATATTGTTTGAAGCCTCCATC |
| *WRKY75-3* | LOC103964033 | GTTTTCCACTTTGTCTCGGTGA | CTGGCTCCTTGTTTGAAATGC |
| *WRKY75-4* | LOC125474253 | TTGCTCCTCATCAGCTAATTATCCA | GCGCAACAACCCATCTGAA |
| *ACT2* | GU830959 | GGACATTCAACCCCTCGTCT | ATCCTTCTGACCCATACCAACC |

**Supplementary Table 2.** Effects of 1-MCP treatment on wax composition proportion at fruit surface in ʻYuluxiangʼ pear during storage.

| Wax compositions proportion  （%） | Storage Time （d） | | | | | | | | |
| --- | --- | --- | --- | --- | --- | --- | --- | --- | --- |
|  | 0 | 7 | | 14 | | 21 | | 28 | |
|  |  | Control | 1-MCP | Control | 1-MCP | Control | 1-MCP | Control | 1-MCP |
| Alkanes | 25.6 | 18.1 | 20.0 | 17.2 | 19.8 | 16.3 | 21.9 | 15.5 | 17.5 |
| Alkenes | 1.1 | 5.9 | 2.7 | 7.7 | 2.5 | 10.7 | 5.0 | 12.1 | 6.4 |
| Fatty acids | 1.6 | 4.7 | 1.3 | 5.4 | 1.4 | 4.8 | 3.2 | 4.6 | 4.6 |
| Esters | 1.8 | 12.5 | 5.2 | 10.9 | 7.6 | 21.6 | 12.2 | 18.1 | 15.5 |
| Aldehydes | 5.6 | 11.6 | 11.6 | 11.5 | 11.4 | 11.3 | 13.2 | 7.7 | 9.6 |
| Primary alcohols | 3.7 | 3.9 | 5.0 | 3.2 | 4.3 | 3.7 | 5.3 | 2.2 | 2.8 |
| Triterpenoids | 51.0 | 35.1 | 45.2 | 35.3 | 46.0 | 24.0 | 32.8 | 29.8 | 35.8 |
